# Natural Products Treat Colorectal Cancer by Regulating miRNA

**DOI:** 10.3390/ph16081122

**Published:** 2023-08-09

**Authors:** Shuoxi Guo, Meiqi Chen, Shuangyang Li, Zijun Geng, Ye Jin, Da Liu

**Affiliations:** School of Pharmacy, Changchun University of Chinese Medicine, Changchun 130117, China; shuoxiguo_1824@163.com (S.G.); 15567958349@163.com (M.C.); lisyang1206@163.com (S.L.); JimmyG_98@163.com (Z.G.)

**Keywords:** colorectal cancer, epigenetic: non-coding RNA, natural product, curcumin, grape seed

## Abstract

Diseases are evolving as living standards continue to improve. Cancer is the main cause of death and a major public health problem that seriously threatens human life. Colorectal cancer is one of the top ten most common malignant tumors in China, ranking second after gastric cancer among gastrointestinal malignant tumors, and its incidence rate is increasing dramatically each year due to changes in the dietary habits and lifestyle of the world’s population. Although conventional therapies, such as surgery, chemotherapy, and radiotherapy, have profoundly impacted the treatment of colorectal cancer (CRC), drug resistance and toxicity remain substantial challenges. Natural products, such as dietary therapeutic agents, are considered the safest alternative for treating CRC. In addition, there is substantial evidence that natural products can induce apoptosis, inhibit cell cycle arrest, and reduce the invasion and migration of colon cancer cells by targeting and regulating the expression and function of miRNAs. Here, we summarize the recent research findings on the miRNA-regulation-based antitumor mechanisms of various active ingredients in natural products, highlighting how natural products target miRNA regulation in colon cancer prevention and treatment. The application of natural drug delivery systems and predictive disease biomarkers in cancer prevention and treatment is also discussed. Such approaches will contribute to the discovery of new regulatory mechanisms associated with disease pathways and provide a new theoretical basis for developing novel colon cancer drugs and compounds and identifying new therapeutic targets.

## 1. Introduction

Cancer remains one of the most alarming health issues humanity faces, and according to information from the National Cancer Database, in 2023, approximately 153,020 people in the United States will be diagnosed with colorectal cancer (CRC), and 52,550 will die from the disease, including 19,550 cases and 3750 deaths in individuals under the age of 50 [1]. Although good results have been achieved for many cancer types with targeted therapies, such as traditional surgical resection, radiotherapy, chemotherapy, immunotherapy, hormonal therapy, molecular targeted therapy, and Chinese medicine, the effective treatment of refractory disease still requires further investigations at the molecular pathological and physiological levels.

CRC begins in the inner layers of the colon, rectum, and appendix and arises due to microsatellite and chromosomal instability [2], the accumulation of various genetic mutations (base substitutions, binding site mutations, and deletions), epigenetic modifications (e.g., non-coding RNA silencing, aberrant DNA methylation, histone acetylation modifications, and chromatin modifications), and gut microbial population changes [3]. Gut microbiota changes cause ecological dysregulation in the body, inducing colon cancer lesions through chronic inflammation. In addition, the sedentary lifestyles of individuals in some Western countries and their poor dietary habits, for example, a lack of fresh vegetable consumption and a high fat and protein diet, have led to an increase in the incidence of CRC worldwide; the risk for familial adenomatous polyposis has also increased [4]. Therefore, it is necessary to find new treatment options and more effective therapies to treat colon cancer to alleviate patients’ suffering and prolong their survival.

Natural products are compounds or small molecules produced from natural and biological resources [5], isolated and extracted from naturally occurring organisms, or formed in animals and plants by biochemical action and photosynthesis [6]. There is increasing evidence that natural products have anti-inflammatory, anti-aging, anti-bacterial, and anti-viral effects on the body in vivo and in vitro and can inhibit tumorigenesis, cancer development, and micro- and macromolecule production [7]. Natural products are also widely available, inexpensive, structurally diverse, rich in biologically active substances, and have few side effects; thus, such products play an important role in the development of novel anti-cancer drugs and lead compounds.

## 2. Biogenesis of miRNA Molecules and Their Mechanism of Action in Tumors

The discovery of ribonucleic acid (RNA) brought about major findings in the field of molecular biology in the second half of the twentieth century, and one of the most important discoveries was that of many types of small RNA molecules (sRNAs), later categorized as non-coding RNAs (ncRNAs), such as tRNAs, rRNAs, uRNAs, snoRNAs, snRNAs, and siRNAs. This group also includes many of the microRNAs (miRNAs) commonly found in living organisms [8]. Ambros et al. discovered the first miRNA, Lin-4, an endogenous regulator of genes that control developmental timing, in 1993 [9].

**Figure 1:**
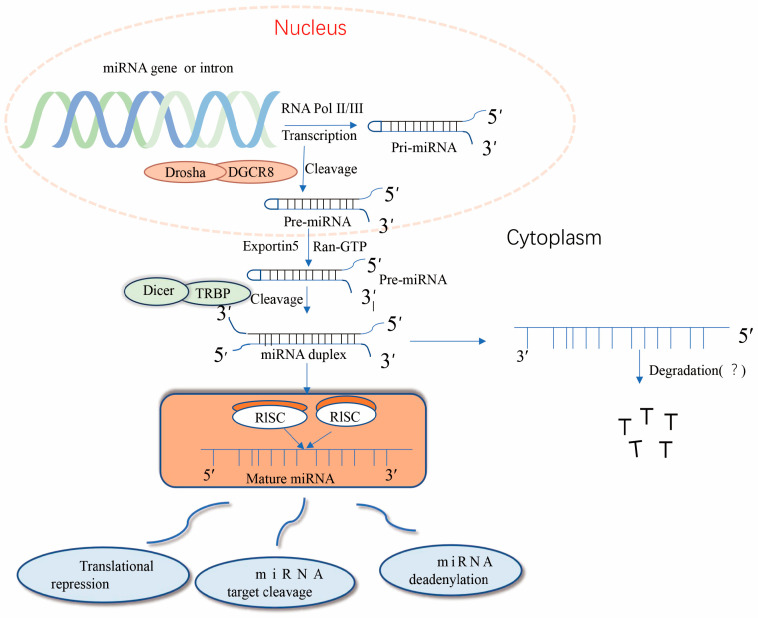
Typical pathway of miRNA formation. miRNA genes are transcribed in the nucleus primarily through the RNA polymerase complex (RNA Pol II) to form hairpin-structured long primary miRNA transcripts (pri-miRNAs). The 5′ pri-miRNA terminus has a guanosine cap, but the 3′ terminus is not always polyadenylated. dgCR8 and the Drosha protein microprocessing complexes direct the processing of pri-miRNAs, leading to the release of the 5′ and 3′ ends of the transcripts and subsequent conformational changes. The resulting precursor miRNAs (pre-miRNAs) are recognized through their 3′ free end and transported to the cytoplasm through the nuclear pore by the Exportin-5 and Ran-GTP complexes. In the cytoplasm, pre-miRNAs are bound by a complex of Dicer and TRBP proteins, and Dicer eliminates the transcript’s ring structure, forming a double complementary strand. The RISC degrades or inhibits miRNA formation; however, some miRNAs are still detected in the cells and bodily fluids of organisms, and their roles remain unclear.

Nuclear miRNA biogenesis begins with the production of primary miRNA transcripts ~300–1000 nucleotides in length by RNA polymerase II or III [10]. Immediately thereafter, nuclear pri-miRNA is cleaved by RNaseIII Drosha into a hairpin structure to form an 80–100-nucleotide intermediate precursor miRNA (pre-miRNA) [11]. After initial shearing, the pre-miRNA is cleaved by the transporter protein esportin-5, a Ran-GTP-dependent dsRNA-binding protein), and the pre-miRNA is transported from the nucleus to the cytoplasm, where it is further cleaved the RNaseIII Dicer enzyme to produce mature miRNA [12]. Together with other proteins, mature miRNAs form RNA-induced silencing complex (RISC), which can degrade or repress target mRNA [13]. Importantly, precursor miRNAs have a hairpin structure instead of a contiguous double-stranded structure; hairpins can be present at the 5′ and 3′, with a higher frequency at the 3′ end [14]. The classical miRNA biosynthesis pathway is shown in Figure 1. miRNA expression dysregulation has been observed in numerous diseases over the past three decades.

Despite significant progress in understanding the basic mechanisms of miRNA biosynthesis, little is known about the mechanisms controlling miRNA biogenesis and how this process is dysregulated in carcinogenesis. According to recent findings, the mechanism of miRNA dysregulation involves the following processes: amplification or deletion of miRNA genes, abnormalities or epigenetic dysregulation of transcription factors, and defects in genes in the miRNA biogenesis pathway [15]. Initially, it was not thought that these tiny RNAs (miRNAs) could have serious consequences for human health or lead to the development and progression of cancer. However, miRNAs have a wide range of gene targets and are involved in various pathological events [16]. The expression of abnormal miRNAs is often involved in carcinogenesis, which must be taken seriously. miRNAs have been classified according to their functions and dysregulation in malignant tumors [17] as tumor miRNAs that target tumor suppressor genes and tumor suppressor miRNAs that target oncogenes and impede their downstream functions [18]. Modifications of the transcriptome of these miRNAs alter the processes of the genes they target, such as cell division, differentiation, angiogenesis, migration, apoptosis, and metabolism [19]. More interestingly, the canonical role of these small non-coding RNAs is to pair with the 3′ untranslated region of the target miRNA and repress miRNA transcription or induction. In addition to playing an extremely important role in the development of various organisms [20], miRNAs cause deficient survival, maturation, and efficacy in NK cells, reinforcing the important function of miRNAs in innate immunity [20]. Meanwhile, natural products, such as curcumin, lignans, rosemary extract, tretinoin lactone alcohol, β-carotene, and bittersweet, have been shown to alter miRNA translation through numerous pathways [21]. Thus, such products could positively or negatively regulate miRNAs and inhibit colon cancer cell growth or induce aberrant apoptosis in CRC therapy [22]. For example, miR-155 targets claudin-1, a dynamic protein associated with genes that regulate various cellular functions, such as proliferation, migration, and epithelial–mesenchymal transition (EMT) [23]. When miR-155 is highly expressed, it can act as a tumor suppressor by regulating the claudin-1 pathway, promoting the migration and invasion abilities of CRC cells [24]. Thus, miRNAs may have diagnostic and prognostic value for CRC patients and are potential targets for CRC gene therapy.

In the following sections, we will highlight the role of miRNAs and natural products in human cancer, analyze their regulatory elements, and discuss the emerging mechanisms of action underlying the natural product-targeted regulation of miRNA and their contribution to cancer pathogenesis.

## 3. The Roles and Mechanisms of Natural Products in the Prevention and Treatment of Colon Cancer

Natural drugs, such as curcumin and bitter ginseng bases, have long been used to prevent and treat cancer [25]. Natural products are associated with different advantages and challenges in comparison with traditional synthetic molecules in the process of drug discovery due to their great scaffold diversity and specific structural complexity [26]. Natural products play important anti-cancer roles in cancer immunotherapy, mainly attributed to their ability to remodel the immunosuppressive tumor microenvironment (TME). Thus, natural products could be an alternative approach for improving immune function in the complex TME [27], which includes cellular (e.g., T cells, B cells, macrophages, myeloid-derived suppressor cells, and cancer-associated fibroblasts) and non-cellular components. TME immune cells are closely associated with sensitivity to therapeutic agents in numerous cancers, including rectal cancer [28].

Tumor-associated macrophages are traditionally divided into two types, the immunosuppressive/anabolic M2 phenotype and the classical inflammatory M1 phenotype; M1 macrophages produce pro-inflammatory cytokines and reactive oxygen/nitrogen species essential for host defense and tumor cell killing [29], while M2 macrophages have anti-inflammatory and angiogenic functions in tumors, blocking tumor growth and migration. In addition, natural products can enable tumor growth and migration and act as microtubule protein stabilizers, inducing cell cycle arrest and acting as tumor suppressors [30]. For example, rhodopsin inhibits tumor growth by inhibiting IRF4, STAT6, and C/EBPb signaling and M2-like polarization [31].

All studies on natural products have indicated that they can affect apoptotic cell death, cell proliferation, migration/invasion, and angiogenesis by modulating the activity or expression of their molecular targets or targeting multiple oncogenic signaling pathways simultaneously [32]. Natural products have also been shown to inhibit metastasis [33]. Therefore, natural products can undoubtedly be used as adjuvants to improve drug sensitization in chemoresistant cancers and enhance therapeutic efficacy, thus reducing patient suffering. It is expected that we will soon better understand natural product-related targets and how to improve their bioavailability to reduce drug-related toxicity and disrupt oncogenic networks in cancer cells [34], enabling the selection of effective natural compounds for different types of tumors.

## 4. Natural Products Can Mediate miRNAs to Promote Apoptosis or Inhibit the Proliferation of CRC Cells

In recent years, many miRNAs have been identified as potential therapeutic regulators of CRC. Apoptosis is an active programmed cellular process that occurs in response to some stimuli and allows cells with damaged DNA to “commit suicide” before they become cancerous; thus, apoptosis is an important basis for tumorigenesis and an effective therapeutic target in cancer [35]. Dysregulation of apoptosis allows malignant cells to escape death, leading to the uncontrolled proliferation of cancer cells and tumor formation [36]. The hypoxic and low nutrient level of the TME may disrupt the cellular homeostasis maintenance function of the endoplasmic reticulum (ER), eventually leading to the accumulation of unfolded proteins and ER stress [37]. However, ER stress is associated with apoptosis in cancer cells; therefore, blocking the adaptive pathway of ER stress or promoting apoptosis is an effective approach to treating cancer. Recently, natural products and their derivatives have been reported to produce potential anti-cancer effects through the ER stress pathway [38]. Piperine produces ROS, CHOP, JNK, and cytochrome c in HT-29 cells, suggesting that the ER stress-mediated apoptosis resulting from piperine treatment is associated with mitochondrial dysfunction [39]. Piperine also inhibits tumor development and progression by inducing apoptosis, suppressing metastasis, enhancing cell cycle arrest, and downregulating other pathways.

Bitter ginseng bases were found to target the mRNA 3′-untranslated region of ERBB3 and MECOM in colon cancer cells, upregulating miR-22. Bittersweet has been suggested to induce apoptosis and G0/G1 cell cycle blockade in colon cancer cells and downregulate the Wnt/β-catenin and MEK/ERK pathways, thereby directly or indirectly exerting therapeutic effects [40]. Rosemary (*Rosmarinus officinalis* L.) extract and its active ingredients have been shown to be natural and potent antitumor suppressors in colon cancer cells.

**Figure 2:**
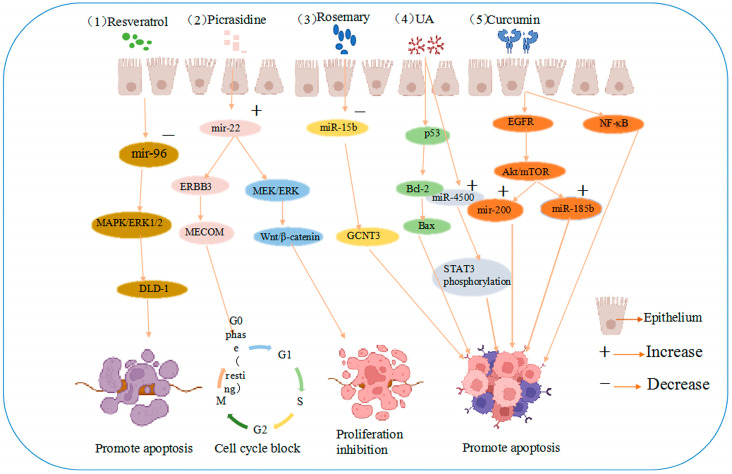
Anti-cancer mechanisms of five agents (1) resveratrol induces growth inhibition and apoptosis in colon cancer DLD-1 cells by reducing miRNA-96 expression through MAPK/ERK1/2 signaling pathway activation. (2) Picrasidine induces apoptosis and G0/G1 cell cycle arrest in colon cancer cells by upregulating miR-22, which targets the mRNA 3′-untranslated region of ERBB3 and MECOM and the Wnt/β-catenin and MEK/ERK pathways. (3) Rosemary extract prevents colon cancer cell metastasis and dysplasia via GCNT3, the target of miR-15b. (4) UA induces apoptosis by regulating Bax and Bcl-2 and upregulating miR-4500 expression in CRC cells through the p53 pathway, inhibiting STAT3 phosphorylation. (5) Curcumin is involved in NF-κB inflammatory process and regulates the Akt/mTOR pathway by downregulating EGFR and oncogenic miR-21 and upregulating mir-200 family translation, promoting apoptosis.

Rosemary extract downregulated miR-15b in SW480 cells, and an electronic analysis predicted that miR-15b targets glucosaminyltransferase 3 (GCNT3), a tumor suppressor [41]. These results suggest that the active extract of rosemary could contribute to the prevention of colon cancer cell metastasis and dysplasia by inducing GCNT3 [42]. The same study revealed that resveratrol is the most effective compound in the stilbene family. Resveratrol is a powerful antioxidant that scavenges DNA-damaging free radicals and inhibits the progression of various cancers [43]. In cellular studies, resveratrol increased antioxidant, anti-inflammatory, and oncogenic inactivating enzyme levels, reduced proliferation, and induced apoptosis in cancer cells [44]. In a miRNA microarray study of SW480 human colon cancer cells injected into mice, the tumors of mice injected with resveratrol demonstrated higher miR-96 levels, thought to be associated with the decrease in the levels of the miR-96 target, KRAS [45]. More importantly, resveratrol can regulate miRNA-96 expression and inhibit tumor progression by activating the MAPK/ERK1/2 signaling pathway to synergistically induce growth inhibition and apoptosis in colon cancer DLD-1 cells, as shown in Figure 2 [46].

Quercetin is found in many plants and foods and is thought to be capable of preventing and treating CRC [47]. Importantly, quercetin can regulate the translation levels of relevant cancer miRNAs, such as the let-7 family, and exerts anti-inflammatory effects by downregulating miR-146a and the NF-κB pathway, inhibiting cancer cell metastasis [48]. Furthermore, treating colon cancer cells with flavonol-containing fractions revealed that the antitumor effect of quercetin is regulated by miR34a, mainly through p53-related pathways [49].

Many other natural products derived from plant seeds, flowers, leaves, and stems have been assessed in different studies and found to help prevent tumorigenesis and development. Curcumin is an orange−yellow crystalline still powder, a natural product isolated from the ginger plant of the family Tennantaceae [50]. Its active constituents have received much attention from the medical community in recent years due to their low toxicity, safety, and beneficial effects on the human body, including anti-aging, anti-cancer, anti-hypertensive, anti-inflammatory, and anti-urinary effects [51]. The anti-inflammatory properties of curcumin are partly attributed to its ability to inhibit COX-2, an enzyme involved in inflammation and the production of inflammatory stimuli, such as nitric oxide synthase, NF-κB, and prostaglandins [52]. Recent studies have revealed that curcumin has antitumor potential since it can regulate miRNAs through epigenetic regulation, for example, decreasing oncogenic miR-21 expression and increasing mir-200 family, let-7 family, and miR-185b expression [53], inhibiting the proliferation or spread of tumor cells. Similarly, curcumin can not only downregulate the translation level of oncogenic miR-21 and its related target genes by specific targeting but also regulate the Akt/mTOR pathway by downregulating EGFR, inhibiting the proliferation of colon cancer cells (Rko and HCT116) and inducing abnormal apoptosis [54].

Similarly, baicalin is commonly used to treat intestinal diseases, such as diarrhea and colon cancer, and a previous study on the antitumor mechanism of baicalin in colon cancer revealed that it induces apoptosis through the miR-217/DDK1-mediated Wnt signaling pathway [55]. In another study, low miIR-491-5p levels were observed in CRC tissues and cell lines at different TNM stages and differentiation states, and baicalin was suggested to inhibit CRC tumor cell growth by targeting and regulating miRNAs [56].

Ursolic acid (UA), a pentacyclic terpenoid, is derived from medicinal plants, such as *C. alba* and *C. purpurea*, and several fruits; UA is an antitumor compound that targets oncogenic proteins and their associated miRNAs [57]. There is growing evidence that the anti-cancer activity of UA is associated with the activation of mitochondria-dependent signaling pathways, including mitochondrial energy metabolism, oxidative stress, and mitochondrial p53-mediated pathways; UA also exhibits pro-apoptotic or anti-proliferative capacities in tumors by regulating the expression of mitochondria-related proteins, such as Bax, Bcl-2, cytochrome c, and cystatin-9 [58]. UA has also been shown to induce apoptosis by inhibiting STAT3 phosphorylation in CRC cells via increased miR-4500 expression; therefore, UA can potentially be used to treat CRC [59].

Lignocaine has been found to inhibit the epithelial-to-mesenchymal transition of CRC cells by inhibiting CREB1 expression [60]. Lignans upregulate miR-384 and downregulate PTN (a growth factor) expression, inhibiting colon cancer tumor development. Thus, unsurprisingly, the anti-CRC effects of lignans are likely mediated in part through the miR-384/PTN axis [61]. In conclusion, the aforementioned natural products are involved in CRC carcinogenesis and development and also demonstrate potential as therapeutic agents.

## 5. Novel Role of Natural Product-Targeted Regulatory miRNAs as Biomarkers in CRC

It is widely believed that CRC development requires a sequence of genetic changes to drive the transformation of normal colonic epithelial tissue to malignant CRC. If patients with colon cancer could be detected and treated before they develop advanced-stage disease, patient mortality rates would subsequently be reduced [62]. Thus, numerous screening methods have been introduced to reduce the incidence of CRC in high-risk groups, such as carcinoembryonic antigen testing, colonoscopy, and occult blood testing. However, these screening methods have some limitations; for example, invasiveness, low specificity, poor population compliance, and a high risk of clinical complications, such as colon perforation in patients with underlying disease. Some methods are also limited by the complex required pre-test preparation [63].

**Figure 3:**
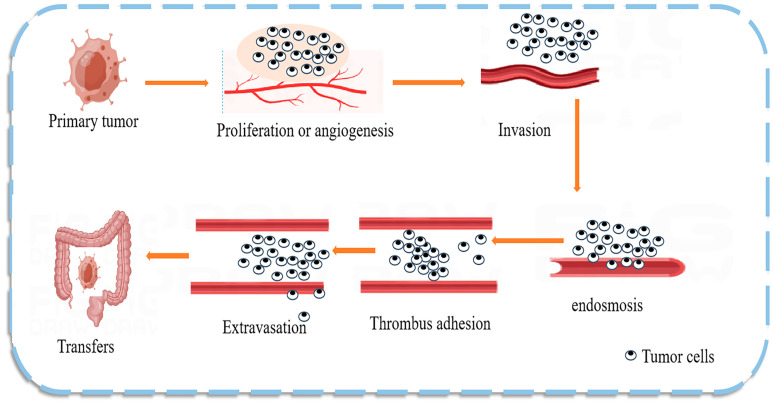
Mechanism of colon cancer tumor metastasis. Tumor metastasis is the main cause of death in patients with malignant tumors. Tumor cells break away from the primary site and invade the extracellular matrix and nearby blood and lymphatic vessels, leading to cell proliferation or angiogenesis. The tumor cells then enter the circulatory system and adhere to the platelets and endothelial cells at the target site, extravasate, and form a tumor at the new site, effectively evading the immune response.

The mechanism of CRC metastasis is shown in Figure 3. Numerous studies have found that natural products can regulate the expression of CRC miRNAs that act as predictive biomarkers. miRNAs can inhibit the translation of oncogenes, participate in cancer development, and play an important role in CRC cells by regulating cell proliferation and differentiation pathways [64]. Dysregulated miRNAs are commonly considered potential diagnostic and prognostic tumor markers and are detectable in stool, serum, plasma, and tissue samples [65]. miRNAs are good biomarkers due to their unique characteristics, such as high tissue specificity, good sensitivity and stability, and ease of detection in body fluids; therefore, the use of miRNAs as prognostic and predictive biomarkers in the therapeutic process is of particular clinical relevance. For example, high circulating blood miR-21 levels are strongly associated with CRC [66], breast cancer, and cancer susceptibility [67]. One interesting study demonstrated that miR-194 could act as a tumor suppressor in CRC by targeting the PDK1/AKT2/XIAP signaling pathway, suggesting that miR-194 is a potential diagnostic marker and therapeutic targe t [68]. The same study also found a set of exosomal miRNAs (including let-7a, miR-1229, miR1246, miR-150, miR-21, miR-223, and miR-23a) in CRC, indicating that miRNA biomarkers are likely common in colon cancer [69].

Another group exposed colon cancer cells to soy extract for 72 h and observed inhibited protein kinase C and cyclooxygenase-2 (COX-2) activity and expression [70]. The density of cancer cells was also significantly reduced after soy extract treatment, suggesting the potential of soy extract in treating colon cancer. Several reports have demonstrated significantly higher levels of miR92 and miR173p in the plasma of colon cancer patients versus those in healthy controls [71]. In contrast, miR-221/202 prevents cancer metastasis by altering signaling pathways and is a potential biomarker for monitoring patient response to treatment [72]. Similarly, miRNA dysregulation is frequently observed in CRC and drives colorectal carcinogenesis. miR-31-3p and miR-31-5p expression levels have been evaluated as noninvasive biomarkers in patients with colon cancer in phase II clinical trials [73].

Given that regulatory miRNAs targeted by natural products are being investigated as potential therapeutic targets, understanding the basis of miRNAs in CRC development and promotion may help to further understand the pathways that can be targeted for cancer prevention. Predicting cancer development and investigating more accurate biomarkers could advance our understanding of the disease and greatly improve patient prognosis and overall survival.

## 6. Natural Products Regulate miRNA Expression and Play Chemopreventive Roles in CRC

Although the prognosis of advanced CRC remains discouraging, the disease is curable in its early stages, highlighting the importance of prevention and early detection. Many drugs and dietary components have demonstrated efficacy in preventing CRC in animal and cellular models, with natural products demonstrating the fewest side effects. Numerous recent studies have revealed that natural products can target various miRNAs, and these miRNAs can also regulate molecular targets that alter disease onset and progression processes. For example, certain natural products can act as chemopreventive agents by upregulating miRNAs that silence proto-oncogenes [74]. Preventive miRNA expression is thought to be regulated in cancer cells through DNA methylation [75]. For example, the hypermethylation of miRNA promoters (let-7, miR-34, miR-342, miR345, miR-9, miR-129, and miR-137) reduces their expression and contributes to cancer development. Decreased miR-143 expression in CRC cells leads to increased methyltransferase activity and cancer cell proliferation [76], as validated by RT-PCR. Five miRNAs (miR-1247-5p, miR-1293, miR-548at-5p, miR-107, and miR-139-3p) are expressed at lower levels in colon cancer patients than in healthy controls, which may be important for cancer progression and beneficial for polyp detection and colon cancer prevention; these miRNAs could also be used to predict tumorigenesis or recurrence [77].

Notably, grape seed extract (GSE), a by-product of commercial grape juice and winemaking processes, contains proanthocyanidin, a flavonoid comprising dimers, trimers, other catechins, and epicatechin oligomers [78]. Proanthocyanidin has preventive effects in breast, head and neck, lung, colon, and other cancers, and miRNA array studies have shown long-term positive regulation of miR-19a, miR-20a, and let-7a and negative regulation of miR-103, miR-135b, miR-148a, miR-196a, and miR-205 in the colonic mucosa of mice with tumors fed GSE. GSE inhibits NF-κB activation and significantly reduces colon tumor size in a dose-dependent manner [79], suggesting that it is a potential natural preventive compound for CRC. In a similar study, it was found that walnuts, which contain high amounts of butyric acid and carob quinone and small amounts of proanthocyanidins and flavonoids, can inhibit colon cancer in a thymus-free nude mouse model subcutaneously injected with HT-29 CRC cells; reduced expression levels of miR-1903, miR-467c, and miR-3068 and increased expression of miR-297a were observed [80]. Natural products inhibit tumor cell proliferation or apoptosis by regulating ncRNA, as shown in Table 1. Researchers have identified many natural products for cancer prevention and treatment, providing new ideas for clinical cancer treatment. Revealing potential epigenetic molecular targets through phytochemicals may advance our fundamental understanding of the relationship between carcinogenesis and phytochemicals and drive the development of chemopreventive or therapeutic strategies.

## 7. Natural Drugs Regulate ncRNA to Modulate Drug Resistance in Tumor Cells

There is growing evidence for aberrant miRNA expression in numerous malignancies, which can also affect chemoresistance. In recent years, natural product active ingredients have been found to play a role in reducing the toxic effects of chemotherapy, improving chemoresistance, and enhancing tumor resistance [93]. Scholars have made extensive attempts to prevent CRC by addressing chemoresistance and increasing the likelihood of successful treatment potential to prevent CRC [94], in particular through identifying the possible mechanisms underlying chemoresistance and increasing the sensitivity of CRC cells to chemotherapy [95].

Emerging studies have shown that almost all colon cancer patients develop drug resistance, which limits the therapeutic effect of anti-cancer drugs, ultimately leading to chemotherapy failure [96]. However, drug resistance in CRC arises through multiple molecular mechanisms, such as reduced drug uptake, increased drug metabolism [97], drug inactivation, DNA damage, irregularities in cell cycle checkpoints, and disturbances in cellular autophagy mechanisms [98]. Drug resistance is the failure of treatment due to the reduced effectiveness of drugs, including antibiotic, antiviral, and chemotherapeutic agents, during the treatment of various diseases [99].

Among multiple ncRNAs, miR-24 was the first chemoresistance-associated miRNA to be identified and was found to promote methotrexate resistance through binding site polymorphisms in the dihydrofolate reductase gene [100]. Traditional chemotherapeutic agents, such as 5-fluorouracil (5-FU), oxaliplatin, and other chemotherapeutic agents [101], are nonselective and remain commonly used, unfortunately leading to frequent adverse side effects such as muscle pain, stomach pain, diarrhea and vomiting, throat pain, and blood abnormalities [102]. Therefore, there is an urgent need to develop new alternative therapeutic strategies. A literature review showed that cancer patients secrete different levels of ncRNA in comparison with normal patients; thus, ncRNAs are potential ideal biomarkers for predicting early disease progression and drug resistance in cancer [103]. The chemotherapeutic agent 5-Fluorouracil (5-FU) is used to treat metastatic CRC worldwide; however, 5-FU resistance has become more prevalent and is an important reason for treatment failure in colon cancer [104]. SchA (isolated from Schisandra chinensis) was assessed in two 5-FU-resistant colon cancer cell lines (HCT116 and SW480); miR-195 expression was high in SchA-treated cells, which reduced cell viability through the inhibition of the PI3K/AKT and NF-κB pathways [105]. It is unsurprising that SchA sensitizes 5-FU-resistant colon cancer cells to 5-FU by upregulating miR-195 since a similar study revealed that compounds isolated from *Cinnamomycetes* spp. (AC), a Taiwanese endemic species that grows as a parasitic fungus in *Cinnamomycetes* hardwoods of the camphor family [106], induce apoptosis in colon cancer cells (HT-29 and SW-480) [107].

**Figure 4:**
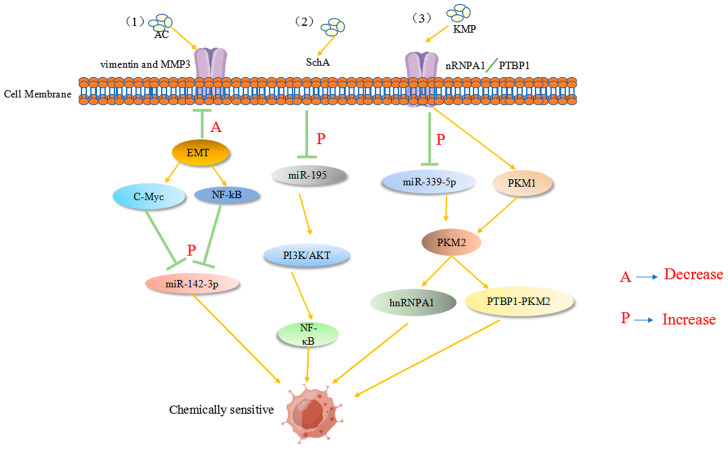
(1) AC acts on tumor-associated proteins, such as MMP3, and enhances 5-Fu chemosensitivity by downregulating the tumor suppressor inflammatory factor C-Myc (A in the figure), upregulating the NF-κB pathway, and increasing miR-142-3p expression (P in the figure). (2) SchA upregulates miR-195 in colon cancer (P in the figure) and acts on the PI3K/AKT and NF-κB pathways to increase 5-Fu chemosensitivity. (3) KMP acts on heterogeneous nuclear ribonucleoprotein A1 (hnRNPA1) and polypyrimidine bundle binding protein (PTBP1) targets to upregulate miR-339-5p expression. The expression of M2-type pyruvate kinase (PKM2) is decreased by miR-339-5p, while PKM1 is stimulated by the direct targeting of hnRNPA1 and PTBP1, enhancing chemosensitivity.

Ant cinnamic acid has been reported to maximize the therapeutic effect in colon cancer patients by upregulating the tumor suppressor inflammatory factor miR-142-3p, thereby downregulating many cancer stem cell-related genes, enhancing 5-FU chemosensitivity, and inhibiting colon carcinogenesis and progression [108]. Previous studies have revealed that miR-155 and membrane-linked protein A2 (ANXA2) play a substantial role in CRC tissues/cells, not only modifying the miR-650/ANXA2 axis via the miR-155 pathway but also enhancing CRC progression and resistance to oxaliplatin via M2 macrophage polarization [109]. Recently, miR-199b-3p expression was found to be considerably upregulated in cetuximab-resistant and sensitive CRC cells. miR-199b-3p downregulation re-established the inhibitory effect of cetuximab on CRC cells, suggesting that miR-199b-3p silencing resensitizes cetuximab-resistant CRC cells to the drug [110]. Another report showed that astragaloside (AS-IV) can inhibit the epithelial–mesenchymal transition in CRC by inducing miR-134 expression, which significantly downregulates the CREB1 (a transcription factor) signaling pathway, thereby improving chemotherapy sensitivity [111]. Kaempferol (KMP), which mechanistically increases miR-339-5p expression, acts on heterogeneous nuclear ribonucleoprotein A1 (hnRNPA1) and polypyrimidine bundle binding protein (PTBP1) targets. Reducing the expression of M2-type pyruvate kinase (PKM2) through miR-339-5p while stimulating PKM1 by directly targeting hnRNPA1 and PTBP1 regulates the miR-326-hnRNPA1/A2/PTBP1-PKM2 axis, which undoubtedly plays an important role in overcoming resistance to 5-Fu therapy [112]. As shown in Figure 4, active ingredients in natural products can reverse the drug resistance process in colon cancer tumor cells. However, to date, obstacles, such as variability in patient characteristics and lack of standardized miRNA assays, have hindered the translation of promising findings into clinical applications. Further studies focused on natural products are expected to involve large clinical trials to explore the underlying mechanisms of CRC and validate the therapeutic, prognostic, and predictive potential of miRNAs regulated by natural products.

## 8. Conclusions

Despite decades of efforts toward preventing and fighting cancer, it remains one of the three deadliest diseases worldwide. Natural products have been described as an irreplaceable source of medical therapies for human oncology because of their multiple pharmacological activities, multi-targeting capabilities, and diverse chemical structures. Over the past three decades, a series of studies have shown that natural products can play an important role in CRC cell proliferation, metastasis, and chemoresistance by indirectly modifying CRC-related signaling pathways, EMT, and angiogenesis by targeting miRNAs. Abnormal miRNA-targeted regulation of proto-oncogene and oncogene expression serves as a biomarker for CRC diagnosis, treatment response assessment, and prognostic evaluation, which provides new clinical ideas for the early diagnosis of CRC patients and could prolong their survival and reduce suffering. In this review, we summarized the anti-CRC effects of some natural products from different sources and their role in TME antagonization and immune regulation; for example, curcumin, lignan, and rosemary extracts can alter the miRNA expression profile and target multiple genes simultaneously. We also discussed some natural products that target and regulate miRNAs that act as potential markers of CRC and drug resistance; these miRNAs inhibit cell migration and metastasis.

Despite the exciting progress that has been made in investigating natural products as modulators of colon cancer therapy in various studies and the broad benefits in miRNA regulation, such as the classical downregulation of cancer cell-associated miRs (e.g., miR-21, the miR-17-92 cluster, and miR-92) enabling tumor suppressor genes (p53, PDCD4, and PTEN), the potential to modulate treatment-resistant cells, and the use of natural products to treat colon cancer through epigenetics, numerous issues remain to be addressed. First and foremost, natural products will demonstrate individual patient and TME differences and could be affected by cancer heterogeneity. Second, a deeper and more comprehensive exploration of the immune system signaling pathways associated with CRC is needed to aid in the selection of more effective natural products. Third, most natural products have a wide range of pharmacological effects; however, their targets and molecular mechanisms associated with tumor immunity have not been fully elucidated. Many studies have found that bioactive compounds modify epigenetics in a dose-dependent manner. Therefore, it is necessary to determine the effective doses and concentrations of these compounds for preventing or treating cancer in the future. Overall, natural products play a major role in the regulation of miRNAs in various cancers, and targeting miRNAs has shown promising therapeutic effects. However, further efforts are required to unravel ncRNA functions and understand their precise mechanisms.

## Figures and Tables

**Table 1:** Natural products inhibit tumor cell proliferation or apoptosis by regulating ncRNA.

Natural Products	ncRNA	Expression	Cell Models	Potential Clinical Values	Type of Bio-marker	Reference
Rosemary Extract	miR-15b	Decline	SW620, DLD-1	Biomarkers	Diagnostics	[81]
Curcumin	miR-17-5p, miR-20a miR-27a, miR-21, miR-130a	Decline	RKO, SW480 HCT116	Biomarkers	Diagnostics	[82]
Walnuts	miR-467c miR-1903, miR-3068	Decline	HT-29	Biomarkers	Prognosis	[83]
GSE	miR-19a, miR-20a, miR-103, miR-135b, miR-148a, miR-196a	Decline	HCT116, SW620	Biomarkers	Prognosis	[84]
HAG	miR-29b	Raise	HCT116, DLD-1, LOVO	Biomarkers	Diagnostics	[85]
*Rosmarinus officinalis* L.	miR-15b	Raise	SW480	Biomarkers	Diagnostics	[86]
Sulforaphane	miR-23b, miR-27b	Decline	NCM460, NCM356	Biomarkers	Diagnostics	[87]
baicalein	miR-23a, miR-30a, miR-31c, miR-151, miR-205a, miR-29	Raise Decline	HCT116, Panc-1, A549	Biomarkers	Diagnostics	[88]
AC	miR-142-3p	Decline	HT-29, SW-480	Biomarkers	Prognosis	[89]
AS-IV	miR-29c	Raise	SW620, HCT116	Biomarkers	Diagnostics	[90]
Spica Prunellae	miR-34a	Decline	HCT-8	Biomarkers	Prognosis	[91]
UA	miR-4500	Raise	HCT-15, HCT-116, HT-29, Caco-2	Biomarkers	Prognosis	[92]

## Data Availability

Data sharing is not applicable to this article.
